# Exploring Differences in Dogs’ and Wolves’ Preference for Risk in a Foraging Task

**DOI:** 10.3389/fpsyg.2016.01241

**Published:** 2016-08-23

**Authors:** Sarah Marshall-Pescini, Ingo Besserdich, Corinna Kratz, Friederike Range

**Affiliations:** ^1^Comparative Cognition Unit, Messerli Research Institute, University of Veterinary Medicine, Medical University of Vienna–University of ViennaVienna, Austria; ^2^Wolf Science CentreDorfles, Austria

**Keywords:** risk-taking, wolves, dogs, feeding ecology

## Abstract

Both human and non-humans species face decisions in their daily lives which may entail taking risks. At the individual level, a propensity for risk-taking has been shown to be positively correlated with explorative tendencies, whereas, at the species level a more variable and less stable feeding ecology has been associated with a greater preference for risky choices. In the current study we compared two closely related species; wolves and dogs, which differ significantly in their feeding ecology and their explorative tendencies. Wolves depend on hunting for survival with a success rate of between 15 and 50%, whereas free-ranging dogs (which make up 80% of the world dog population), are largely scavengers specialized on human produce (i.e., a more geographically and temporally stable resource). Here, we used a foraging paradigm, which allowed subjects to choose between a guaranteed less preferred food vs. a more preferred food, which was however, delivered only 50% of the time (a stone being delivered the rest of time). We compared identically raised adult wolves and dogs and found that in line with the differing feeding ecologies of the two species and their explorative tendencies, wolves were more risk prone than dogs.

## Introduction

Human and non-human animals are often faced with decisions which may involve taking risks. Humans are mostly risk-averse, so for example when asked to choose between two containers, one with 10 Euros for certain vs. the other with a 50/50 chance of containing 20 Euros or being empty, subjects will mostly choose the safe option (Kahneman and Tversky, 1979). Studies on risk-taking in non-human species have focused mostly on primates, where results have been more controversial: cotton-top tamarins, lemurs, and bonobos have been shown to be risk averse, whereas chimpanzees, macaques, and capuchin monkeys have been shown to be risk prone (McCoy and Platt, 2005; Heilbronner et al., 2008; Stevens, 2010; Haun et al., 2011; Rosati and Hare, 2012, 2013; De Petrillo et al., 2015) and common marmosets have been shown to be risk neutral (Stevens, 2010). However, the tendency for being risk prone seems to depend on several factors, including how the choices are presented, energy budget, individual differences and the ecology of the species.

When faced with the risk of a potential loss, both humans and a number of non-human species have been shown to increase their risk-preference, whereas if the same choices are framed in a context of a potential gain, the preference for risk taking can be reversed (humans: Tversky and Kahneman, 1981; starlings: Marsh and Kacelnik, 2002; capuchin monkeys: Chen et al., 2006; Lakshminarayanan et al., 2011). This tendency for risk-preferences to reverse depending on whether the choice is framed as a loss or a gain is known as the ‘reflection effect’ or ‘loss aversion’ and is one of the primary predictions of Prospect Theory (Kahneman and Tversky, 1979).

Energy budget has also been shown to affect risk-taking, with individuals in a positive energy budget state being more risk averse than an individuals in a negative state (Stephens, 1981). Furthermore, in several species, variation in risk-taking behavior has been associated with individual differences in boldness and aggression (mice: Blaszczyk et al., 2000; cichlid fishes: Brick and Jakobsson, 2002) and a genetic predisposition for risk-taking in great tits was shown to be positively associated with a greater inclination for explorative behaviors (van Oers et al., 2004). The ecology of a species has also been consistently associated with risk-taking preferences. A comparison between three sympatric species of tits revealed that risk-preference was directly affected by their feeding ecology. Indeed the more insectivorous species were shown to be risk prone whereas the more granivorous species was risk averse (Kawamori and Matsushima, 2012). Comparative work across primate species further supports the link between feeding ecology and risk preference. For example, chimpanzees, that depend more heavily on patchy fruit trees and invest time in hunting where outcomes are often uncertain (Gilby and Wrangham, 2007), have been shown to be more risk-prone (Heilbronner et al., 2008; Haun et al., 2011) and more willing to wait for a delayed reward (Rosati et al., 2007) than bonobos, that rely more heavily on terrestrial vegetation, a more temporally and spatially reliable food source (Wrangham and Peterson, 1996).

In the current study, we addressed whether a species’ ecological environment may shape its risk preference by comparing wolves and dogs in a foraging task. Wolves and dogs are interesting to study in this context because they diverged only between 15000 and 30000 years ago (Wang et al., 2013). Wolves and free-ranging dogs (i.e., 75–80% of the world dog population; Lord et al., 2013) show similar behavioral patterns in terms of living in pack-like groups and forming stable hierarchical structures (Mech and Boitani, 2003; Bonanni et al., 2010; Cafazzo et al., 2010). Crucially for the present study, however, they differ conspicuously in their foraging strategies. Wolves rely predominantly on hunting with success rates ranging between 10 and 50% (Mech et al., 2015), whereas free-ranging domestic dogs, although capable of preying on wild species (e.g., Manor and Saltz, 2004; Young et al., 2011; Silva-Rodriguez and Sieving, 2012), rely mostly on human generated resources (Butler et al., 2004; Atickem et al., 2009; Vanak and Gompper, 2009a; Newsome et al., 2014), leading most researchers to conclude that free-ranging dogs can be considered scavengers specialized in exploiting human refuse (Schmidt and Mech, 1997; Butler and du Toit, 2002; Vanak and Gompper, 2009b). This is supported by the occurrence of key mutations to specific genes associated with the digestion of starch during domestication (Axelsson et al., 2013), indicating a direct adaptation to the new dietary opportunities offered by dogs’ association with humans.

Considering wolves’ higher reliance on hunting behavior compared to dogs’ scavenging on distributed and more stable food sources, we hypothesized that, in the feeding context, wolves’ would show a higher preference for making risky choices than dogs. Furthermore, in a recent study comparing identically raised wolf and dog pups, we found that the former showed a higher inclination to explore both a new environment and a novel object (Marshall-Pescini et al., submitted). Considering that great tits’ risk-taking has been positively associated with a higher propensity for explorative behavior (van Oers et al., 2004), our finding regarding the higher explorative tendencies of wolf compared to dog pups would also support predictions suggesting that wolves will be more risk-prone than dogs.

To test the risk preference of wolves and dogs, we adapted a method previously used with apes (Heilbronner et al., 2008; Rosati and Hare, 2012, 2013) in which animals are presented with a two-way choice task. One option results in a ‘safe outcome’ where animals always obtain a less preferred food item (i.e., a dry food pellet). The alternative choice represents the ‘risky outcome’ where on 50% of trials subjects obtain a preferred food item (i.e., a piece of meat), but in the remaining 50% they obtain a non-edible item (a stone). Before each trial, subjects are shown the potential outcome of each choice, so they are aware of the ‘odds’ of the risky vs. safe outcome. The same task was presented to comparably raised and kept wolves and dogs.

## Materials and Methods

### Subjects

Overall seven wolves (three females, four males; mean age in years: 4.7, range: 2.1–6.1) and seven mixed-breed dogs (five females, two males: mean age in years: 3.2, range: 2.9–3.8) housed at the Wolf Science Centre successfully completed the task. An additional two wolves (both males) and two dogs (both males) had to be excluded because they either failed to pass the initial training phase (one wolf, one dog: 11 sessions of food visible) or developed a side preference (one dog), or was too wary of the apparatus and would not approach the task at all (one wolf).

Wolves and dogs at the WSC^1^ are raised and kept in the same way, and participate in various behavioral tests every week, where they are rewarded with the same food items used in the current test (i.e., dry food, sausage, meat, and chicks). All wolves and dogs are worked in separation from their pack members on a daily basis and participation in all training and testing sessions are voluntary (i.e., animals are called out from their packs but they can choose to join the testing enclosure or not at their own discretion). Food items used in the current study are equally familiar to both wolves and dogs and used only in training/testing situations. Aside from food received during training/testing, animals are also fed in their home enclosures in a group setting. The frequency of feeding in the group context is not constant since it largely depends on the amount of food animals received during training/testing on any given day. The type of food delivered in the group setting is somewhat different for wolves and dogs. Wolves are predominantly fed rabbit carcasses and dogs receive a mixture of meat and dry food pellets of a different quality from those used for training/testing. No food deprivation regime was employed prior to testing, animals were tested during the morning or afternoon having been fed the night before. For more details relating to the upbringing and keeping of the animals, please see Range and Virányi (2013a,b).

### Ethical Statement

No special permission for use of animals (wolves) in such socio- cognitive studies is required in Austria (Tierversuchsgesetz 2012— TVG 2012). The relevant committee that allows running research without special permissions regarding animals is: Tierversuchskommission am Bundesministerium für Wissenschaft und Forschung (Austria). Ethical approval was obtained from the ‘Ethik und Tierschutzkommission’of University of Veterinary Medicine (Protocol number 06/02/97/2014).

### Apparatus

**FIGURE 1:**
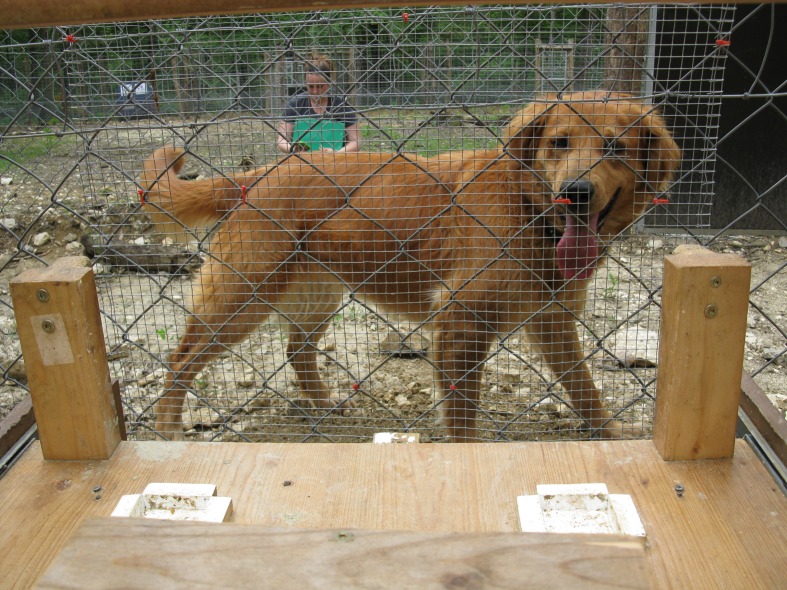
**The apparatus: two wooden blocks (targets), were placed adjacent to the food locations (white squares).** When the table-top was slid forward animals could touch one of the targets with either nose or paw, which resulted in the researcher delivering the food item from the corresponding white square.

The apparatus consisted of a 49 cm × 57 cm movable table-top with two wooden blocks (15 cm × 5 cm) fixed 42 cm apart on opposite sides of the tray (**Figure 1**). Each wooden block (henceforth ‘target’) was immediately adjacent to the location in which the food was placed. The apparatus was placed in front of the animal’s enclosure in such a way that the target became available to the animal only when the table-top was moved forward by the experimenter, and hence the wooden target slotted through the fence lines. Touching the target with either nose or paw corresponded to a choice by the animal to obtain the food placed in the corresponding location. Food was delivered to the animal by placing the chosen food item on a delivery rod, placed under the table top, which could be slid forward through the fence line and into the animal’s enclosure.

**FIGURE 2:**
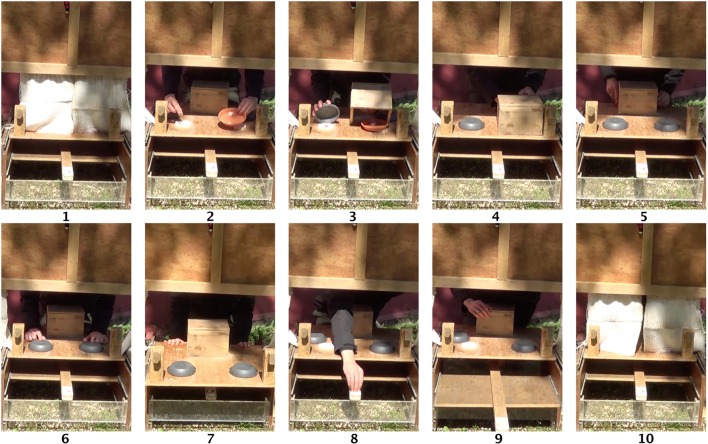
**Pictorial description of procedure from the animal’s perspective: (1) the curtain is closed, (2) the food outcomes are presented (the potential outcomes of the risky choice are both shown to the animal in the ‘outcome container,’ i.e., the brown dish), (3) the dish and barrier are placed over the food items, (4) behind the barrier the experimenter selects the appropriate outcome for the risky option unseen by the subject, (5) the identical overturned containers are presented, (6) both containers are touched simultaneously, (7) the tray is pushed forward to allow animals to choose, (8,9) the food is placed in the delivery slide and pushed forward, (10) the curtain is closed thereby ending the trial**.

The experimenter’s face and body were invisible to the subject since a wooden panel was placed above the table-top, and screens were placed on either side of it. The animals could only see the experimenter’s hands (see **Figure 2** for the animal’s view of the apparatus). Considering wolves’ and dogs’ sensitivity to human social cues, this setup was considered essential to guarantee the animal’s choices were not affected by the experimenter’s inadvertent cuing. A second person standing quietly 4 m away, and who could not see which side was baited as the ‘risky vs. safe option’ called out which side the animal chose, allowing the experimenter to deliver the corresponding item.

### Overall Procedure

Five phases were conducted. Two phases (i.e., the food visible and food invisible training) served to introduce animals to the apparatus and how it functioned. A third phase consisted of a food preference test carried out to establish the individual’s preference for different food types. Following these preparatory phases, a risk introduction phase was presented consisting of ‘exposure sessions’ in which animals were familiarized with the differing outcomes of safe and risky choices by presenting one of these possibilities at a time, and ‘comprehension sessions’ in which the animals’ understanding of the task contingencies was evaluated. Finally, in the fifth and final phase, four test sessions were presented, in which animals could choose between the risky outcome or safe outcome. In these sessions, additional ‘attention trials’ were conducted to ensure that animals were paying attention to the task on a trial-by-trial basis. Each subject was individually tested and no more than one session was conducted per day.

### Testing Procedure

All trials began with the experimenter opening the curtain to reveal the table-top. Subjects then saw the experimenter simultaneously placing the least preferred food type (i.e., dry food) in one food location (the safe option; always dry food) and the ‘risk outcome’ container in the other food location (the risky option; **Figure 2.2**). In test trials, the risk outcome container held one piece of the preferred food type (i.e., meat/sausage/chick) and one stone. Because the dry food pellet was a standard size (1.5 cm squared, Royal Canin, professional, German Shepard Adult), the preferred food, whatever the type, was cut to be of the equivalent size (weight of dry food pellet: 4 g, sausage: 7 g, meat: 7 g). Animals had approximately 5 s to look at the options presented, then the experimenter covered both sides simultaneously (**Figure 2.3**), the safe option was covered by a small gray dish (17 cm in diameter, 3 cm tall) and the risky option was covered by a small free- standing box (22 cm wide, 18 cm deep, 15 cm tall) which acted as a visual barrier whilst the experimenter placed the correct outcome item for the risky option under an identical gray dish. The box was then removed, leaving animals with the option to choose between two identically looking containers (**Figures 2.4,2.5**). At this point, the risky option only contained one of the possible outcomes that the subject had previously seen in the risk outcome container, and animals did not know which, before making a choice. Finally, the experimenter touched both overturned containers simultaneously (**Figure 2.6**) and pushed the table-top forward so that the animals could choose one of the options, by touching with their nose to the corresponding target (**Figure 2.7**). Hence, subjects always knew what they could receive from the safe option, but did not know whether they would receive a good or bad outcome from the risky option. If the animal chose the safe option, the experimenter did not reveal the contents of the risky option. Once the animal touched the target, the experimenter placed the corresponding food item on the delivery rod and pushed it forward (**Figures 2.8,2.9**). The trial ended when the animal either took the food or walked away (when the stone was delivered). To signal the end of the trial, the curtain was closed (**Figure 2.10**), allowing the experimenter to prepare the next trial unseen.

In all trials, subjects had 20 s to choose once the table-top was pushed forward; if they failed to choose within this time interval, the trial was repeated at the end of the session. If an animal did not participate for three consecutive trials (in whatever phase), the session was stopped and repeated on the following test day. Only complete sessions were included in the data analyses.

### Phases

#### Phase 1- Food Visible Training

There were 24 consecutive trials per session. Animals were trained to touch the target corresponding to the side on which the food was placed. Only one piece of food was presented at a time, placed either on the right or left food locations on the table-top (in a semi-randomized order- not more than two times in a row on one side). Once the food was visibly placed in position, the experimenter pushed the table-top forward so the animals could touch the target with their nose to obtain the food reward. If the incorrect target was chosen, no food was delivered, if the correct target was chosen, food was placed on the delivery rod and given to the animal. Sessions were repeated until the animals reached the criteria of 18/24 correct choices in a single session. Dry food pellets randomly alternated with equivalent sized pieces of sausage were used during training.

#### Phase 2- Food Invisible Training

There were 24 consecutive trials per session (preceded by four ‘reminder’ trials identical to the food-visible training described above). As in the previous training, only one piece of food was presented at a time either on the right or left food location (semi-randomized as above). The experimenter then covered both locations with identical overturned containers (to introduce this aspect of the task) and pushed the tray forward so the animal could choose which target to touch. The experimenter then lifted the container on the side that the animal had chosen showing what was underneath (food or nothing). If the incorrect target was chosen, no food was delivered, if the correct target was chosen food was placed on the delivery rod and given to the animal. The same food types were used as above and again sessions were repeated until the animal reached the criterion of 18/24 correct choices in a single session.

#### Phase 3- Food Preference Test

There were 24 consecutive trials per session. Two pieces of different food types were presented in the two food locations. The least preferred vs. most preferred food type were established for each animal (dry food pellets were always the low value reward, which was paired with one of the following high value items of equal size than the dry food: meat/beef, sausage, or chick). Choice of food combinations for each animal was based on data from previous food preference tests conducted at the WSC. However, to ensure the preference was still the same, the combinations were presented again, and if necessary changed, until animals reached the criterion of 18/24 trials choosing the preferred food in a single session.

#### Phase 4- Risk Introduction Session

It consisted of (a) ‘exposure sessions’ and (b) ‘comprehension sessions.’ Comprehension sessions were delivered to the animals interspersed amongst exposure sessions in the following sequence: Exposure sessions 1–4: Comprehension session 1; Exposure sessions 5–7: Comprehension session 2; Exposure sessions 8–10: Comprehension session 3. A final Comprehension session (session 4) was presented after all test sessions were conducted.

(a) Twenty-one *exposure trials* were presented per session. In these trials animals only had one option available at a time: either the ‘safe option’ or the ‘risky option’ presented in one food location, whereas the other food location remained empty. The ‘risky option’ was presented exactly in the same way as in subsequent test trials- described above in the ‘general test session procedure.’ When the risky option was presented, the animals received the preferred outcome on half of trials, whereas they received the non-edible item (a stone of the same size as the food pieces) on the other half. Outcomes were semi-randomized with never more than two of the same outcomes in a row. In the first session of exposure trials animals were first presented with seven safe trials followed by 14 risky trials to facilitate learning of the contingencies. In subsequent sessions (2–10) 14 risky and seven safe trials were presented in a semi-randomized order (i.e., never more than two trials of the same type in a row). The location of the risky vs. safe option was also randomized across trials.

(b) Twenty-four *comprehension trials* per session were conducted to assess the comprehension of the task contingencies (**Table 1**). Two types of comprehension trials were presented to the animals in a semi-randomized order (never more than two trials of the same type in a row):

**Table 1:** Food distribution in the safe and risky option in comprehension and test sessions.

Conditions in comprehension and test trials	Safe option	Risky option (50%)
Comprehension trials Type A	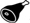 + 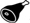	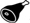 / 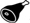
Comprehension trials Type B	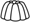	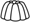 / 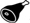
Test trials	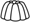	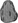 / 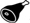
Attention 1 trials	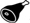	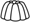 / 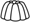
Attention 2 trials	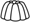	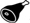 / 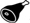


*In the Risky option animals see both items in the ‘outcome container’ but receive only one. Legend: 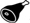 = meat; 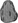 = stone; 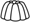 = dry food.*

*Comprehension a* (12 trials). Here, the safe option as well as the risky option consisted of two pieces of the preferred food. If, after the exposure trials, subjects understand that the risky option only provides one of the possible outcomes that they previously saw in the outcome container, they should choose the safe option. That is, even though the same amount of food is initially presented in both the safe container and the risky outcome container, the safe option will ultimately provide more food.

*Comprehension b* (12 trials). The safe container contained only one piece of dry food, and the risky outcome container contained one piece of dry food and one piece of the preferred food. If subjects actively compare the potential reward they could receive from the safe and risky options, they should prefer the risky option because it delivers a piece of preferred food in 50% of the trials, and on the other half the dry food (which they would obtain anyway if they chose the safe option).

Hence, overall, in the risk introduction session animals received a total of 210 exposure plus 72 comprehension trials. Comprehension trials were deemed important to assess the animal’s understanding of the basic rule underlying the task (i.e., that the risky option only delivered one food item previously seen in the outcome container). However, to avoid overly increasing the number of pre-test trials, no criteria was set in the exposure trials to move on to the comprehension trials and neither was a criteria set to move on from the comprehension trials to the test sessions after completing the risk introduction sessions. Rather the animal’s performance in comprehension trials was then used in the analyses to ascertain its effect on the choice of the risky option in test sessions (see below).

#### Phase 5- Test Session

Four 26-trial sessions were presented. Each test session consisted of 20 test trials (safe vs. risky option) and six attention trials (**Table 1**). Three attention trials were presented at the beginning of the session, and the remaining three attention trials were presented after the last test trial. A total of 80 test trials and 24 attention trials were hence presented to each animal.

*Attention 1* (three trials in each test session). These trials were presented in each test session to confirm that animals were attending to the available reward on a trial-by-trial basis. In these trials, the safe option provided one piece of preferred food, whereas in the risky container there were two pieces of a non-preferred food. Subjects should choose the safe option because it provides the preferred food type.

*Attention 2* (three trials in each test session). The safe option provided the non-preferred foods, whereas in the risky container there were two pieces of preferred food. Subjects should choose the risky option because it provides the preferred food type.

A final set of 24 comprehension trials (12 *comprehension a*+ 12 *comprehension b*) were presented after all the test trials were conducted.

Overall the procedures were adapted from Heilbronner et al. (2008) and Rosati and Hare (2012, 2013). The main setup was similar to that used with chimpanzees in Heilbronner et al.’s (2008) study; however, because dogs’ numerical competence is somewhat limited (Range et al., 2014), we chose to adopt Rosati and Hare (2012) method of using foods of different value to the animals to assess their preference for risk-taking. However, whereas, used three different types of food, which varied on the animal’s preference scale, we only used two and introduced a non-edible item as the lowest outcome. This method was adopted because from previous (unpublished) data on food preference in wolves and dogs at the Wolf Science Centre, whereas we could firmly establish a constant preference between two types of food (i.e., dry food vs. meat), finding a third food type, which was constantly a middle preference proved very difficult (both for wolves and dogs).

### Analyses

All tests were video-recorded. The animal’s choice was noted on paper during testing and inter-observer reliability (by a second coder) was carried out on 20% of data from videos. Agreement on which target was chosen by the animals was 100%.

To evaluate the animals’ understanding of the task prior to testing, and their preference for risky choices during testing, a one-sample *t*-test was carried out (having ascertained test assumption were met) to assess whether both dogs and wolves were above chance level in each comprehension and test session. Furthermore, for each comprehension and test session each individual’s performance was evaluated separately with a binomial test. To further investigate the animal’s understanding of the task, we carried out the same analyses also separating the comprehension trials by type (see Supplementary Material). Moreover, we compared wolves’ and dogs’ performance in each comprehension session using a generalized linear mixed model (GLMM), with binomial distribution, with correct choices (1/0) as the dependent variable, and species and session as the independent factors. Similarly, a GLMM was run to compare wolves’ and dogs’ choices. Hence, risky choices (vs. safe ones, 1/0) and correct (vs. incorrect, 1/0) choices in attention trials were entered as dependent variables in two separate analyses with species and session as the independent variables. In all models the subject’s identity was inserted as a random factor. To evaluate whether the animals’ performance in comprehension sessions and attention trials was correlated with their choice of a risky outcome in test trials we ran a Spearman’s correlation test for dogs and wolves separately. To assess whether the good vs. bad outcome of the prior risky trial affected the subsequent choice of a risky option, we ran a GLMM with risky choice (vs. safe choice 1/0) as the dependent variable and the outcome of the risky choice in the previous trial as the independent variable (good/preferred food vs. bad/stone outcome 1/0).

## Results

### Training Trials

On average, wolves took four sessions to reach criterion in both the food visible and food invisible conditions. Similarly, dogs took on average four sessions to reach criterion in the food visible condition and three sessions in the food invisible condition (see **Supplementary Table S1**, for the number of sessions required to reach criterion by each individual).

### Food Preference

All wolves showed a significant preference for one food type over the other one in their first session (binomial test: 18 choices or above out of 24, *p* = 0.02), with the only exception of one subject that showed a significant preference only in the second session. In contrast, dogs took between one and six sessions before showing a significant preference for one food type over the other. Two dogs required six sessions, and one dog five sessions, before showing a clear preference for one food type over another (N. of sessions for each dog: 1,6,6,5,2,3,2). Given the large variability in the number of sessions required to establish dogs’ food preferences, we analyzed whether this factor (i.e., number of sessions to criteria in the preference test) affected the frequency with which dogs chose the risky option in test trials; however, this was not the case (glmm: *z* = 0.346, *p* = 0.729).

### Comprehension Trials

Both dogs and wolves performed above chance level in all comprehension sessions (Wolves: session 1: 70%, session 2: 79%, sessions 3 and 4: both 85%; Dogs: session 1: 63%, session 2: 67%, session 3: 63%, session 4: 66%; all *p* < 0.05, see **Table 2**).

**Table 2:** Wolves’ and dogs’ performance in comprehension trials.

Species	Session 1	Session 2	Session 3	Session 4
Wolves (*n* = 7)	17 ± 1.2 (70%);	19 ± 1.1 (79%);	20.57 ± 0.72 (85%);	20.7 ± 0.8 (85%);
	*t* = 4, *p* = 0.007	*t* = 6.3, *p* = 0.001	*t* = 11.92, *p* < 0.001	*t* = 10.8; *p* < 0.001
Dogs (*n* = 7)	14.9 ± 0.5 (63%);	16.57 ± 0.84 (67%);	15.14 ± 0.96 (63%);	15.9 ± 1.4 (66%);
	*t* = 5.6, *p* = 0.001	*t* = 5.4, *p* = 0.002	*t* = 3.27, *p* = 0.017	*t* = 2.7, *p* = 0.036


*Mean ± SE for correct choices are indicated in each cell alongside the test statistics comparing performance to chance level.*

At the individual level, in session 1, three wolves but no dogs performed above chance level (binomial test significant 18/24, *p* = 0.02). In session 2, five wolves and three dogs performed above chance level. In session 3 (i.e., the last comprehension session before test trials) all seven wolves, but only one dog, performed above chance level. In the final comprehension trials (after test sessions), all wolves and three dogs performed above chance (**Table 3**).

**Table 3:** Individual scores in each comprehension session for wolves and dogs.

Name	Species	Comprehension session			
		
		1	2	3	4
Aragorn	Wolf	17	*23*	*23*	*22*
Chitto	Wolf	13	15	*20*	*20*
Geronimo	Wolf	*19*	*20*	*21*	*21*
Kaspar	Wolf	*19*	*20*	*20*	*23*
Shima	Wolf	13	*20*	*18*	*18*
Tala	Wolf	*22*	15	*19*	*23*
Yukon	Wolf	16	*20*	*23*	*18*
Binti	Dog	15	16	15	*18*
Bora	Dog	17	14	11	10
Layla	Dog	13	16	16	13
Meru	Dog	14	*18*	14	*18*
Nia	Dog	16	*18*	14	16
Nuru	Dog	14	*20*	17	*19*
Zuri	Dog	15	14	*19*	*21*


*Total number of trials per session was 24; highlighted in *italics* scores, which were significantly above chance level.*

**FIGURE 3:**
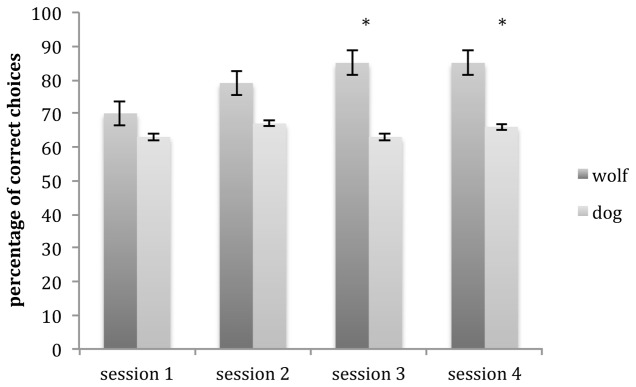
**Mean number of correct choices (and SE) carried out by wolves and dogs in comprehension trials for each session.** Wolves outperformed dogs in sessions 3 and 4. ^∗^*p* < 0.001.

A GLMM showed an interaction between species and session (glmm: *z* = 2.75, *p* = 0.006). Wolves outperformed dogs in session 3 (glmm: *z* = 4.73, *p* < 0.001) and session 4 (glmm: *z* = 3.16, *p* < 0.001) but there was no difference in session 1 (glmm: *z* = 1.85, *p* = 0.065) and session 2 (glmm: *z* = 1.65, *p* = 0.099; **Figure 3**).

For detailed results on performance in each comprehension trial type both at the group and individual level (**Supplementary Tables S2** and **S3**; **Supplementary Figures S1** and **S2**).

### Attention Trials

On attention trials, wolves performed above chance level in all test sessions (all sessions *p* < 0.01, see **Table 3**), whereas dogs performed above chance level in all test sessions except session 4 (sessions 1–3, *p* < 0.05; see **Table 4**).

**Table 4:** Wolves’ and dogs’ performance in attention trials.

Species	Session 1	Session 2	Session 3	Session 4
Wolves (*n* = 7)	5.43 ± 0.2 (90.5%);	5.29 ± 0.36 (88%);	5.29 ± 0.42 (88%);	5.29 ± 0.36 (88%);
	*t* = 2.02, *p* < 0.001	*t* = 6.36, *p* < 0.001	*t* = 5.43, *p* = 0.002	*t* = 6.36, *p* < 0.001
Dogs (*n* = 7)	4 ± 0.3 (67%);	4.43 ± 0.57 (74%);	4.71 ± 0.52 (78.5%);	3.43 ± 0.37 (57%);
	*t* = 3.24, *p* = 0.02	*t* = 2.5, *p* = 0.047	*t* = 3.29, *p* = 0.01	*t* = 1.16, *p* = 0.29


*Mean ± SE for correct choices are indicated in each cell alongside the test statistics comparing performance to chance level.*

The GLMM showed a main effect of species (glmm: *z* = 3.97, *p* < 0.001), with wolves outperforming dogs, but no main effect of session (glmm: *z* = 0.79, *p* = 0.42) and no significant interaction (glmm: *z* = 0.55, *p* = 0.58; see **Supplementary Table S4**; **Supplementary Figure S3**).

### Test Trials

Wolves chose the risky option in 70–95% of test trials compared to dogs that chose this option in 38–76% of trials (see **Table 5**).

**Table 5:** Number of risky choices in each session, as well as total number (and percent) of risky choices made over all test sessions by each individual for wolves and dogs.

Wolves	N. risky options (tot trials = 20)	% risky option	Dogs	N. risky options (tot trials = 20)	% risky option
Aragorn	19,18,19,20	19 (95%)	Binti	11,18,17,15	15.25 (76%)
Chitto	12,13,17,14	14 (70%)	Bora	11,11,1,12	8.75 (44%)
Geronimo	11,17,17,17	15.5 (78%)	Layla	11,10,9,11	10.25 (51%)
Kaspar	19,17,19,17	18 (90%)	Meru	13,16,15,15	14.75 (38%)
Shima	16,14,16,16	15.5 (78%)	Nia	8,14,10,11	14 (70%)
Tala	16,19,16,17	17 (85%)	Nuru	10,16,12,11	12.25 (61%)
Yukon	20,20,20,16	19 (95%)	Zuri	12,18,14,11	13.75 (69%)
Mean		84%			58%


At the group level, dogs chose the risky option significantly above chance in two out of the four test sessions (i.e., sessions 2 and 4, *p* < 0.05, see **Table 6**), whereas wolves chose the risky option above chance in all test sessions (all sessions: *p* < 0.01, see **Table 6**).

**Table 6:** Wolves’ and dogs’ performance in test trials.

Species	Session 1	Session 2	Session 3	Session 4
Wolves (*n* = 7)	16.14 ± 1.3 (80%);	16.86 ± 0.96 (84%);	17.71 ± 0.6 (88.5%);	16.71 ± 0.7 (83.5%);
	*t* = 4.6, *p* = 0.004	*t* = 7.1, *p* < 0.001	*t* = 2.7, *p* < 0.001	*t* = 9.9, *p* < 0.001
Dogs (*n* = 7)	10.86 ± 0.59 (54%);	14.71 ± 1.2 (73.5%);	11.14 ± 1.9 (56%);	12.29 ± 0.7 (61.4%);
	*t* = 0.4, *p* = 0.2	*t* = 3.9, *p* = 0.008	*t* = 0.6, *p* = 0.6	*t* = 3.2, *p* = 0.02


*Mean ± SE for risky choices are indicated in each cell alongside the test statistics comparing performance to chance level.*

At the individual level, six of the seven wolves preferred the risky option above chance in at least three out of four sessions (i.e., chose the risky option on 15 or more trials out of 20 in each session, binomial test, *p* = 0.04) and the remaining wolf showed a preference for the risky option in two of the four sessions. Two dogs preferred the risky option above chance in three out of four sessions, in one session two dogs chose the risky option above chance. One dog performed at chance level in all sessions, and the last dog performed at chance level in all but one session in which it showed a significant preference for the safe option (i.e., chose the risky option only once in 20 trials; **Table 5**).

**FIGURE 4:**
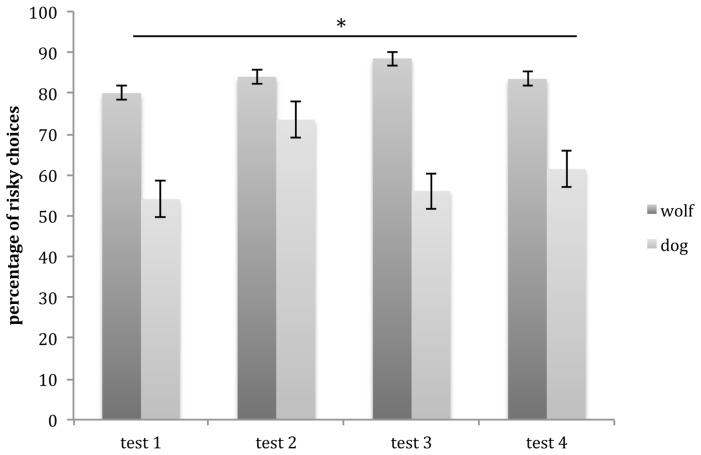
**Mean number of risky choices (and SE) carried out by wolves and dogs in test sessions.** Wolves performed significantly more risky choices than dogs across all test trials. ^∗^*p* < 0.001.

The GLMM showed a main effect of species (glmm: *z* = 4.023, *p* < 0.001), with wolves choosing significantly more risky options than dogs, but no main effect of session (glmm: *z* = 0.95, *p* = 0.34) and no significant interaction (glmm: *z* = 0.406, *p* = 0.68; **Figure 4**).

### Performance in Comprehension and Attention Trials and Choice of the Risky Option

Within species, we looked at whether there was a correlation between the animal’s performance in attention and test trials, and comprehension and test trials. No correlation emerged between the overall performance in attention trials and the number of risky choices in test trials in wolves (*N* = 7, ρ = 1.78, *p* = 0.36); however, this could be do to a ‘ceiling effect’ since wolves’ performance was extremely high in both these trial types. A significant and positive correlation between performance in attention trials and the number of risky choices in test trials emerged in dogs (*N* = 7, ρ = 0.455, *p* = 0.015). No significant correlation occurred between the overall scores obtained in comprehension trials and the number of risky choices in test trials in dogs (*N* = 7, ρ = 0.205, *p* = 0.3) or in wolves (*N* = 7, ρ = 0.34, *p* = 0.074).

Since overall dogs’ performance in attention trials correlated with the number of choices of the risky option in test trials, we ran a comparison between wolves and dogs considering only test sessions in which animals chose correctly in at least five out of six attention trials (i.e., sessions in which dogs’ performance in attention trials suggested they were focused on the choices presented to them). This analysis resulted in the inclusion of two test sessions per dog, and a minimum of two and maximum of four sessions per wolf (total: 10 test sessions for five dogs; 23 session for seven wolves). Overall wolves carried out significantly more risky choices than dogs (glmm: *z* = 2.35, *p* = 0.017; Bonferroni corrected: significant if *p* < 0.017)^2^.

A further comparison was carried out between another subset of dogs and wolves. Whereas in the comprehension session just prior to testing (session 3), only one dog performed above chance level but in the final comprehension session after testing (session 4) three dogs did so, it is possible that at least some dogs acquired an understanding of the task during testing. Hence, to further insure that the species difference was not a mere product of the dogs’ lower understanding of the contingencies of the task, we compared the performance of only those three dogs that performed above chance level in comprehension session 4 with that of wolves. Moreover, to further guarantee that the comparison was made at a stage in which these three dogs understood the task, we compared the wolf-dog performance only in test session 4 (i.e., the last test sessions, carried out right before their above chance level performance in comprehension session 4). Results showed that wolves chose the risky option significantly more than dogs (glmm: *z* = 0.3.29, *p* = 0.001, Bonferroni corrected: significant if *p* < 0.017).

### Alternative Strategies: Olfaction and Previous Outcome

To check that animals were not using olfactory cues to select the ‘risky’ option only when meat was hidden under the outcome container rather than the stone, we looked at the percentage of risky trials in which the animals did in fact obtain the meat. Wolves selected the risky option in a total of 471 trials and of these they obtained the meat in 231 trials (49% of trials). Dogs selected the risky option in a total of 353 trials, and of these they obtained the meat in 183 trials (51.8% of trials). This confirms that animals were not using olfactory cues to determine whether to choose the risky vs. safe option.

To check whether the previous outcome (dry food, stone or preferred food) affected the subsequent choice of the animals (risky vs. safe), we carried out a separate model for wolves and dogs with risky vs. safe choice as the dependent variable, and the previous outcomes as explanatory factors. Previous outcome did not affect the likelihood of an animal choosing the risky option in the subsequent trial for either wolves (glmm: dry food vs. meat: *z* = 0.86, *p* = 0.4; dry food vs. stone: *z* = 1.4, *p* = 0.2; meat vs. stone *z* = 0.729, *p* = 0.5) or dogs (glmm: dry food vs. meat: *z* = 0.5, *p* = 0.6; dry food vs. stone: *z* = 1.3, *p* = 0.2; meat vs. stone *z* = 0.85, *p* = 0.4).

## Discussion

Whereas wolves made between 70 and 95% risky choices in test sessions, dogs made between 38 and 76% risky choices. Indeed wolves consistently chose the risky option significantly more than dogs in all test sessions. Hence wolves, that rely mostly on hunting, show a higher preference for risk as measured in the current study than dogs that rely more on scavenging, suggesting that the different ecological environments (and foraging strategies in particular) of wolves and dogs may have affected their preference/aversion for risk. The present results are in line with studies showing that more insectivorous tits are more risk-prone than more granivorous ones (Kawamori and Matsushima, 2012), and that chimpanzees that rely more on seasonally fruiting trees are more risk-prone than bonobos that depend on more stable terrestrial vegetation (Heilbronner et al., 2008). Taken together, these findings suggest that the less reliable and more transient the staple food source is, the more a species may be willing to take risks.

On average, wolves showed a noticeably high preference for risk (80%). Indeed, their level of risk-preference was comparable to that observed in chimpanzees (65–70%: Heilbronner et al., 2008; Rosati and Hare, 2013) and higher than that reported for bonobos (40–32%: Heilbronner et al., 2008; Rosati and Hare, 2013). Dogs, on the other hand, chose the risky option on average 58% of the time, which was higher than bonobos but not chimpanzees. However, although we largely based our study on Heilbronner et al. (2008) and Rosati and Hare (2012, 2013), results are difficult to compare directly since the odds of the risky option among studies differed. Indeed, whereas in our study the risky odds entailed a preferred food vs. no-food (a stone), in the Heilbronner et al. (2008) study apes were given different quantities of the same preferred food (hence the safe option consisted of four grape halves and the risky option would deliver either seven or one grape halves). In Rosati and Hare (2012) procedure food quality was used similarly to our own setup; however, three food types differing in value were used and there was no potential non-edible outcome. Hence compared to both these two setups, our own entailed a greater potential loss, since it could result in obtaining a non-edible item.

Wolves’ risk-proneness was, however, consistently higher than that of dogs, potentially pointing to the former’s dependence on hunting as the major influencing factor. The results of the comparison between wolves and dogs in the task, however, need closer scrutiny. The assessment of the animals’ aversion or preference for risk in the current paradigm hinges on their understanding of the basic rule governing the task (i.e., that only one of the two items observable in the outcome container was then delivered, whereas whatever item was shown on the ‘safe’ side would always be made available if chosen).

Performance in comprehension trials of type A is particularly relevant here, since animals were presented with two pieces of preferred food in both locations. Hence, unless there was an understanding of the underlying rule (i.e., that only *one* item from the risky outcome container would be delivered), the animals should show no preference for either location. Indeed, results of the separate analyses show that whereas the majority of wolves performed consistently above chance level from the second session on, dogs showed a much more varied performance (see **Supplementary Tables S2** and **S3**; **Supplementary Figures S1** and **S2**). Instead, comprehension type B trials (in which a single dry food pellet in the ‘safe’ location is contrasted with a piece of meat and dry food pellet in the outcome container) could be ‘solved’ based on an immediate strategy of choosing the location where the preferred food item was shown. However, this simple preference (or lack of inhibitory control) is not sufficient to account for the above chance performance in type ‘A’ comprehension trials, since in that case the perceptual features of the two locations were identical. Considering comprehension trials as a whole, dogs’ performance, particularly in session 3 just prior to testing, was significantly worse than the wolves’ performance, and although at the group level dogs performed above chance (suggesting some comprehension of the task), only one dog consistently chose the correct options. In contrast, all seven wolves performed above chance level in the comprehension session prior to testing, suggesting that they had firmly understood the basic rule of the task.

Nonetheless, in the course of the four test sessions, three dogs did begin to understand the contingencies of the task, since they consistently chose the correct option in the final comprehension session. The significant difference in performance of these three dogs and the wolves in the very last test session suggests that wolves were more risk prone than dogs. Similarly, we compared wolves’ and dogs’ performance taking into account only those sessions in which individuals showed a high performance in attention trials. Even in this case the species difference was maintained. Hence, although results in comprehension and attention trials suggest that the task was at the limit of the dogs’ abilities, the selected comparisons between wolves and dogs still resulted in a higher preference for risk in wolves.

A number of other possibilities may explain the wolf-dog difference. First, it could be that over the course of testing, wolves and dogs received a different proportion of high-value reward. However, this was not the case since wolves received the food on 49% and dogs on 52% of trials (see Supplementary Material). This shows that both species experienced similar reward histories during testing and also that animals could not detect which outcome they would receive from the risky option before making their choice (e.g., by using olfactory cues).

Another possible explanation for the dogs’ more varied performance in test sessions is that they followed a different strategy based on the outcome of previous trials. To check for this possibility, we analyzed whether the outcome of the previous trials (i.e., whether the animal had received dry food, a stone, or the preferred food) affected the likelihood of their choosing the safe or the risky option in the subsequent trial. However, this was not the case for either dogs or wolves (see Supplementary Material).

Yet another potential aspect affecting results in the current study is the relative magnitude of the preference for meat over dry food in the two species. Results show that wolves’ preference for meat was evident for most individuals from the first preference session onward, whereas, the preference for meat in dogs was slower to be established with three individuals needing between five and six sessions to show a consistent preference for one of the two foods. Based on these results it could be argued that the ‘magnitude’ of the preference for meat is different in wolves and dogs (potentially also in line with their differing feeding ecologies, given wolves’ higher dependence on hunting). If the value given to meat is higher, the loss involved would also be greater, which may increase the tendency to take risks. This aspect requires further investigation; however, it is important to note that we found no relationship between the number of sessions needed to establish a food preference and the frequency of choosing the risky option in test trials, suggesting that at the individual level this variable did not seem to directly affect the animal’s decision-making process.

An alternative explanation for the wolf-dog difference is that wolves’ bias toward the risky option can be explained by a failure to inhibit an inherent tendency to choose the higher quality reward. In primates, behavioral inhibition (i.e., blocking an impulsive or prepotent response in favor of a more appropriate alternative) has been shown to be affected by both the social dynamics of a species (Amici et al., 2008), their foraging ecology (Stevens et al., 2005; MacLean et al., 2014) and task contingencies (Boysen and Berntson, 1995; Vlamings et al., 2006). In particular the latter studies showed that in a reversed contingency task in which individuals had to choose a smaller amount of food to obtain the larger amount as reward, the bigger the numerical/size difference between the two options the harder it was for animals to inhibit their prepotent response to reach for the larger reward. However, only one study has so far been carried out comparing wolves’ and dogs’ capacity for behavioral inhibition and results showed wolves outperforming dogs in one task and the opposite pattern of results emerging in the second (Marshall-Pescini et al., 2015). No significant correlations between risk-preference and measures of inhibition emerged for the 13 animals (seven dogs and six wolves) that performed all tests in both studies.

Considering dogs and wolves at the Wolf Science Center are raised and kept in the same manner, we conclude that the differences observed are likely due to the different feeding ecologies between the two species. Wolves’ ‘feast-or-famine’ existence (Mech et al., 2015) characterized by significantly riskier foraging situations, may have selected for a more risk-prone set of decision rules. Results are consistent with previous studies comparing chimpanzees and bonobos (Heilbronner et al., 2008; Haun et al., 2011), and closely related tits with differing feeding ecology (Kawamori and Matsushima, 2012), which converge in suggesting that the more transient and less reliable the staple food source of a species, the more likely they will show a preference for risk. Wolves and dogs are, to our knowledge, the first predator and scavenger species tested on a risk-foraging task, and considering the results, it suggests that further evaluation of predators and scavengers may provide interesting insights into which aspects of a species’ feeding ecology may affect preference for risk.

## Author Contributions

SM-P conceived, designed and coordinated the study, analyzed the data and drafted the manuscript. FR and IB helped designing the study. FR participated in data analyses and helped draft the manuscript. IB and CK collected the data and did the initial statistical analyses. All authors gave final approval for publication.

## Conflict of Interest Statement

The authors declare that the research was conducted in the absence of any commercial or financial relationships that could be construed as a potential conflict of interest.

## Footnotes

1 www.wolfscience.at

2 The whole dataset was used once for main analyses, but subsets of it were used again for a further two analyses (once including only animals performed well in attention trials, and a second times using the subset of animals passing criteria in comprehension trials) hence the new alpha level was set at *p* = 0.017 based on Bonferroni.
