# Supplementary material for: Exploring Differences in Dogs’ and Wolves’ Preference for Risk in a Foraging Task
Source: Front Psychol. 2016 Aug 23;7:1241. doi: 10.3389/fpsyg.2016.01241 (PMC4993792; doi:10.3389/fpsyg.2016.01241)
Supplement: Supplementary file 1 [file Data_Sheet_1.DOCX]

***Supplementary Material***

**Exploring differences in dogs’ and wolves’ preference for risk in a foraging task**

**Sarah Marshall-Pescini*, Ingo Besserdich, Corinna Kratz, Friederike Range**

*Correspondence: sarah.marshall@vetmeduni.ac.at

***Supplementary Results***

*Food visible and Food invisible training*

| Name | Species | Food Visible | | Food Invisible |  |
| --- | --- | --- | --- | --- | --- |
| Aragorn | Wolf | 6 | 4 | | |
| Chitto | Wolf | 4 | 3 | | |
| Geronimo | Wolf | 2 | 1 | | |
| Kaspar | Wolf | 6 | 10 | | |
| Shima | Wolf | 5 | 6 | | |
| Tala | Wolf | 8 | 4 | | |
| Yukon | Wolf | 1 | 2 | | |
| Binti | Dog | 3 | 1 | | |
| Bora | Dog | 7 | 3 | | |
| Layla | Dog | 3 | 4 | | |
| Meru | Dog | 10 | 3 | | |
| Nia | Dog | 1 | 1 | | |
| Nuru | Dog | 3 | 4 | | |
| Zuri | Dog | 3 | 5 | | |

**Table S1.** Number of sessions required to reach criterion for each individual in Food visible and Food invisible condition training conditions.

*Comprehension by trial-type*

*Comprehension A*: two preferred food items are placed both in the safe and risky locations. If animals understand that they will only receive one item from the risky outcome container they should choose the safe side. 12 trials were conducted in each session hence binomial significance at the individual level is 10/12 (p=0.038).

In Session 1 at the individual level only one wolf and one dog performed above chance. However, as a group both wolves and dogs performed above chance (wolves: mean=8, SE=0.7, t= 2.9, p=0.027, df=7,; dogs: mean=8.4, SE=0.4, t=6.6, p=0.001, df=7).

In Session 2 at the individual level two wolves and one dog performed above chance, whereas as a group both wolves and dogs were above chance (wolves: mean=9.14, SE=0.7, t= 4.7, p=0.003, df=7,; dogs: mean=8.72, SE=0.56, t=4.8, p=0.003, df=7,).

In Session 3 at the individual level six wolves and three dogs performed above chance and both species performed above chance at the group level (wolves mean=10.14, SE=0.3, t=15.9, p<0.001, df=7,; dogs: mean=8.3, SE=0.7, t=3.4, p=0.015, df=7,).

In Session 4 at the individual level all seven wolves and two dogs performed above chance but only wolves performed above chance at the group level (wolves: mean=10.71, SE=0.3, t=16.5, p<0.001, df=7,; dogs: mean=7.7, SE=0.9, t=1.8, p=0.127, df=7,). For all the above analyses see Table S2 and Figure S2.

| Name | Species | Comprehension Session | N. correct (tot=12 trials) |
| --- | --- | --- | --- |
| Aragorn | Wolf | 1, 2, 3, 4 | 8, 11, 11, 11 |
| Chitto | Wolf | 1, 2, 3, 4 | 9, 12, 10, 11 |
| Geronimo | Wolf | 1, 2, 3, 4 | 7, 8, 9, 10 |
| Kaspar | Wolf | 1, 2, 3, 4 | 8, 9, 10, 12 |
| Shima | Wolf | 1, 2, 3, 4 | 5, 8, 10, 10 |
| Tala | Wolf | 1, 2, 3, 4 | 11, 7, 10, 11 |
| Yukon | Wolf | 1, 2, 3, 4 | 8, 9, 11, 10 |
| Binti | Dog | 1, 2, 3, 4 | 8, 6, 7, 9 |
| Bora | Dog | 1, 2, 3, 4 | 8, 9, 6, 4 |
| Layla | Dog | 1, 2, 3, 4 | 7, 9, 7, 7 |
| Meru | Dog | 1, 2, 3, 4 | 9, 9, 10, 6 |
| Nia | Dog | 1, 2, 3, 4 | 10, 8, 8, 7 |
| Nuru | Dog | 1, 2, 3, 4 | 8, 11, 9, 12 |
| Zuri | Dog | 1, 2, 3, 4 | 9, 9, 11, 9 |

**Table S2.** Individual scores in each comprehension session for A-trials (i.e. two preferred food types in the safe and in the risky location) for wolves and dogs.

**Figure S1.** Mean correct response of wolves and dogs in Comprehension trials A (two preferred food items in safe and risky container-> should go for safe) for each session.

*Comprehension B*: one piece of dry food is placed in the safe container whereas the risky outcome container holds one piece of dry food and one piece of the preferred food. If animals understand the contingencies of the task they should choose the risky side since it allows them to obtain the preferred food on some occasions. 12 trials were conducted in each session hence binomial significance is 10/12.

In Session 1 at the individual level three wolves but no dogs performed above chance. Indeed also as a group only wolves performed above chance (wolves: mean=9, SE=1, t=2.9, p=0.026, df=7; dogs: mean=6.43, SE=0.48, t=0.9, p=0.4, df=7).

In Session 2 at the individual level five wolves and 2 dogs performed above chance, but still as a group only wolves performed above chance level, although a trend emerged in dogs (wolves: mean=9.57, SE=1.2, t=2.9, p=0.026, df=7; dogs: mean=7.8, SE=0.8, t=2.2, p=0.066, df=7).

In Session 3 at the individual level, five wolves but no dogs performed above chance. Indeed also as a group only wolves performed above chance (wolves: mean=10.43, SE=0.6, t=7.2, p<0.001, df=7; dogs: mean=6.8, SE=0.7, t=1.2, p=0.27, df=7).

Finally in Session 4 at the individual level four wolves and one dog performed above chance, although as a group both species performed above chance (wolves: mean=10, SE=0.6, t= 6.5, p=0.001, df=7; dogs: mean=8.14, SE=0.8, t=2.7, p=0.037, df=7). For all the above analyses see Table S3 and Figure S3.

| Name | Species | Comprehension Session | N. correct (tot=12 trials) |
| --- | --- | --- | --- |
| Aragorn | Wolf | 1, 2, 3, 4 | 9, 12, 12, 11 |
| Chitto | Wolf | 1, 2, 3, 4 | 4, 3, 10, 9 |
| Geronimo | Wolf | 1, 2, 3, 4 | 12, 12, 12, 11 |
| Kaspar | Wolf | 1, 2, 3, 4 | 11, 11, 10, 11 |
| Shima | Wolf | 1, 2, 3, 4 | 8, 10, 8, 8 |
| Tala | Wolf | 1, 2, 3, 4 | 11, 8, 9, 12 |
| Yukon | Wolf | 1, 2, 3, 4 | 8, 11, 12, 8 |
| Binti | Dog | 1, 2, 3, 4 | 7, 10, 8, 9 |
| Bora | Dog | 1, 2, 3, 4 | 9, 5, 5, 6 |
| Layla | Dog | 1, 2, 3, 4 | 6, 7, 9, 6 |
| Meru | Dog | 1, 2, 3, 4 | 5, 9, 4, 8 |
| Nia | Dog | 1, 2, 3, 4 | 6, 10, 6, 9 |
| Nuru | Dog | 1, 2, 3, 4 | 6, 9, 8, 7 |
| Zuri | Dog | 1, 2, 3, 4 | 6, 5, 8, 12 |

**Table S3.** Individual scores in each comprehension session for B-trials (i.e. one piece of dry food on the safe side and one piece of dry food + a piece of preferred food in the risky outcome container) for wolves and dogs.

**Figure S2.** Mean correct response of wolves and dogs in Comprehension trials B (one dry food in the safe and one dry food + a piece of meat in the risky-> should go for risky) for each session.

*Attention trials*

| Name | Species | Session | N. correct (tot=6 trials) |
| --- | --- | --- | --- |
| Aragorn | Wolf | 1, 2, 3, 4 | 6, 4, 6, 5 |
| Chitto | Wolf | 1, 2, 3, 4 | 5, 6, 5, 4 |
| Geronimo | Wolf | 1, 2, 3, 4 | 6, 5, 6, 4 |
| Kaspar | Wolf | 1, 2, 3, 4 | 5, 6, 6, 6 |
| Shima | Wolf | 1, 2, 3, 4 | 6, 4, 3, 6 |
| Tala | Wolf | 1, 2, 3, 4 | 5, 6, 5, 6 |
| Yukon | Wolf | 1, 2, 3, 4 | 5, 6, 6, 6 |
| Binti | Dog | 1, 2, 3, 4 | 4, 6, 4, 5 |
| Bora | Dog | 1, 2, 3, 4 | 3, 4, 3, 4 |
| Layla | Dog | 1, 2, 3, 4 | 3, 2, 3, 3 |
| Meru | Dog | 1, 2, 3, 4 | 4, 6, 5, 3 |
| Nia | Dog | 1, 2, 3, 4 | 5, 3, 6, 3 |
| Nuru | Dog | 1, 2, 3, 4 | 4, 5, 6, 4 |
| Zuri | Dog | 1, 2, 3, 4 | 5, 5, 6, 2 |

**Table S4** Individual scores in attention trials during test sessions for wolves and dogs.

**Figure S3.** Mean number of correct choices (and SE) carried out by wolves and dogs in attention trials presented in each test session. Wolves outperformed dogs across all sessions p<0.001.
